# Traumatic ischemic injury in a top of the basilar distribution: a case report

**DOI:** 10.1186/s12883-021-02207-7

**Published:** 2021-04-27

**Authors:** Mihir Gupta, Anudeep Yekula, David Barba, J. Scott Pannell, Jeffrey Tomlin

**Affiliations:** 1 Department of Neurosurgery, University of California San Diego, La Jolla, 1335 Sunset Cliffs Boulevard, San Diego, CA 92107 USA; 2 Department of Neurosurgery, Massachusetts General Hospital, Boston, MA USA; 3 Department of Neurosurgery, Naval Medical Center Portsmouth, Portsmouth, VA USA

**Keywords:** Top of the basilar, Traumatic brain injury, Case report

## Abstract

**Background:**

Top of the basilar syndrome is a rare, heterogeneous disorder that has previously only been described in the setting of acute ischemic stroke in predominantly elderly patients. We present the first reported case of traumatic brain injury (TBI) causing ischemia in a top of the basilar distribution.

**Case presentation:**

A 19-year-old woman suffered an acute subdural hematoma and sustained hypoxemia after being struck by a motor vehicle. Neurosurgical evacuation of the hematoma was undertaken. Magnetic resonance imaging revealed ischemic injury in the midbrain and diencephalic structures fitting a top of the basilar distribution. No associated vascular injury was identified. The patient was eventually discharged in a state of persistent unresponsive wakefulness.

**Conclusions:**

Ischemia in a top of the basilar distribution may occur in the setting of TBI. A high degree of clinical suspicion is required to identify this disorder. Further study of the complex inflammatory microenvironment and associated tissue perfusion dynamics in TBI are needed in order to elucidate the mechanisms underlying ischemic injury patterns, develop management paradigms and predict prognosis.

## Background

Top of the basilar syndrome has been described in small cohorts of adult patients in the setting of stroke. We present a unique case of traumatic brain injury causing ischemic injury in a top of the basilar distribution, and discuss the findings in the context of the emerging understanding of cerebrovascular pathophysiology associated with traumatic brain injury (TBI).

## Case presentation

**Fig. 1:**
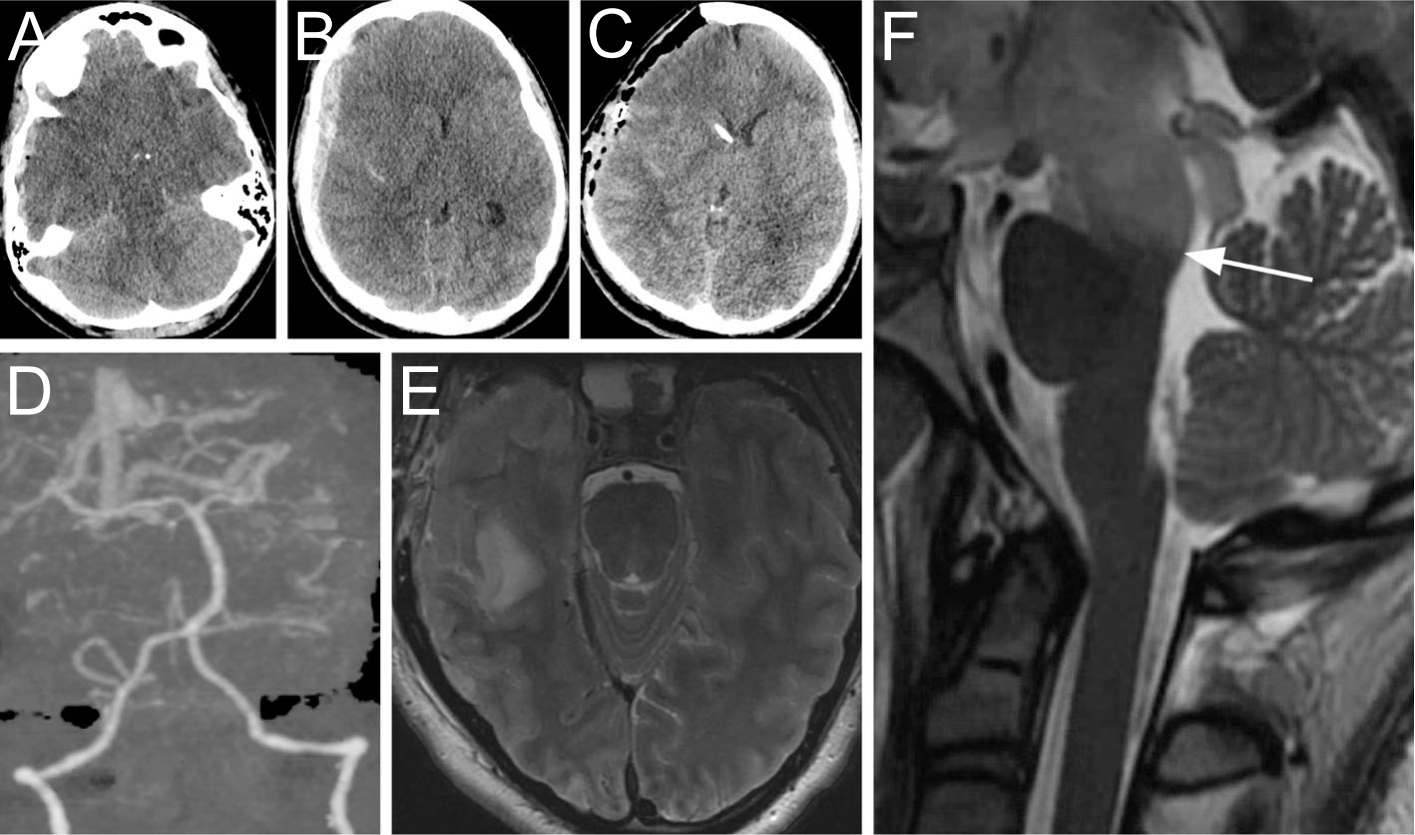
**a, b** Axial noncontrast head computed tomography (CT) scan on presentation showing effacement of the basal cisterns (**a**) and a right convexity acute subdural hematoma causing midline shift (**b**). **c** Axial CT scan immediately following right hemicraniectomy and ventriculostomy placement showing relief of mass effect and evacuation of hematoma. **d** Three-dimensional CT angiography reconstruction showing patent posterior arterial and deep venous circulation. **e, f** T2-weighted magnetic resonance axial (**e**) and sagittal (**f**) images of the cervical spine 18 h postoperatively. The flow void of the basilar artery is visualized at the mid-pons, and a right temporal contusion is evident (**e**). The sharp transition at the pontomesencephalic junction (arrow) highlights hyperattenuation of the midbrain and diencephalon (**f**), with sparing of the remainder of the brainstem

A 19-year-old woman was found unconscious after being struck by a motor vehicle. Upon arrival to our facility, she was hypotensive and unresponsive to noxious stimuli with agonal breathing and fixed, dilated pupils. Noncontrast computed tomography (CT) of the head revealed an acute subdural hematoma causing significant mass effect (Fig. 1). Emergent decompressive hemicraniectomy and ventriculostomy placement were undertaken. Postoperatively the patient’s pupils remained dilated and unreactive. Spontaneous eye opening and breathing as well as corneal, cough and gag reflexes were present. Stimulation elicited decorticate posturing of the extremities. Magnetic resonance imaging (MRI) showed hyperattenuation in the midbrain and diencephalon, sparing the remainder of the brainstem (Fig. 1). No cerebrovascular abnormalities were noted on CT angiography.

**Fig. 2:**
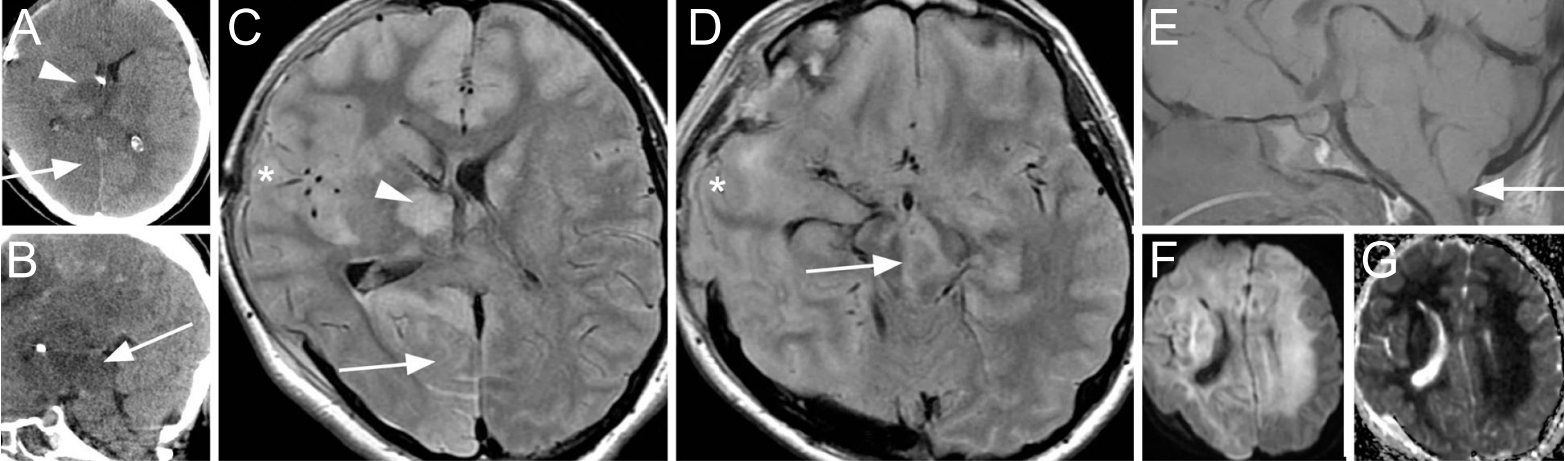
**a, b** Noncontrast head CT axial (**a**) and sagittal (**b**) images two days postoperatively showing hypodensities in a ‘top of the basilar’ injury distribution, affecting the thalami (A, arrowhead), right PCA territory (A, arrow) and midbrain (B, arrow). **c, d** Axial FLAIR MRI sequences ten days postoperatively showing evolution of the ischemic injuries to the thalami (C, arrowhead), right PCA territory (C, arrow), and midbrain (D, arrow). Herniation of the brain through the craniectomy defect (C and D, asterisks) and downward through the foramen magnum (E, arrow) suggests intracranial hypertension. **f, g** Axial diffusion-weighted sequence (**f**) and corresponding apparent diffusion coefficient map (**g**) show diffuse acute supratentorial deep white matter cytotoxic edema

Although intracranial pressure (ICP) was initially well-controlled, she developed sustained refractory intracranial hypertension starting two days postoperatively. CT scan disclosed evolution of the deep ischemic injury, fitting a ‘top of the basilar’ distribution involving the bilateral thalami and midbrain as well as the right posterior cerebral artery (PCA) territory (Fig. 2). MRI on post-injury day 10 confirmed evolution of the infarctions in these regions. The additional development of diffuse supratentorial cytotoxic edema suggested further ischemic injuries and ongoing intracranial hypertension (Fig. 2). The patient required tracheostomy and gastrostomy placement, as well as treatment of central panhypopituitarism. She was eventually discharged to a care facility in a condition of persistent unresponsive wakefulness.

## Discussion

Occlusion of the rostral basilar artery has previously been described in the setting of strokes due to large- or small-artery disease, cardioembolism, or unknown causes. Flow may be obstructed in branches of the basilar artery apex supplying the midbrain, as well as PCA branches supplying the diencephalon and temporo-occipital regions. The protean manifestations of top of the basilar syndrome include disturbances of consciousness, oculomotor palsies, weakness, ataxia, and sleep-wake cycle impairment, frequently leading to poor long-term outcomes [1].

Interestingly, we did not find any direct or indirect evidence of arterial injury or thrombosis such as dissection, occlusion, dense basilar sign or cervical spine injury. Furthermore, although the patient sustained severe TBI, stigmata such as duret hemorrhages and diffuse axonal injury were absent. The reasons the ischemic injury localized to the distal basilar territory are thus unclear. Because the top of the basilar syndrome has not previously been described in pediatric patients, young adults, or the setting of trauma, the pathophysiology, symptoms and outcomes in these populations have not been characterized.

Seminal early studies established hypotension and hypoxia as independent predictors of poor outcome from severe TBI, likely due to secondary ischemic brain injury [2]. Ischemic brain injury remains the leading cause of death and associated with poor outcome following severe TBI. Cerebral blood flow (CBF) alterations associated with TBI remain poorly understood. Dramatic reductions in CBF occur within 24 h after injury, while CBF values from post-injury day two onwards are highly variable and take several weeks to normalize. Ischemia and hyperemia may coexist due to uncoupling between tissue metabolism and blood flow in the early period [3].

Studies of severe TBI have also demonstrated diffuse cerebral cellular hypoxia in the absence of structural abnormalities, overt ischemia, macrovascular injury, or elevated intracranial pressure. This finding is likely due to metabolic and inflammatory derangements that affect the diffusion of oxygen and rate of oxygen consumption, and also cause endothelial swelling, perivascular edema and microthrombosis; this cascade of events leads to microvascular or ‘diffusion barrier’ ischemia [4].

We speculate that the etiology of the initial ischemic injury in our patient was likely a combination of hypoperfusion due to systemic hypotension and reduced CBF due to intracranial hypertension. Additional possibilities such as direct vascular compression, vasospasm, and diffusion barrier ischemia cannot be ruled out. The relative contribution of each of these mechanisms remains unknown.

The development of diffuse, progressive cytotoxic edema even after successful evacuation of the subdural hematoma in our case also bears mention. Cytotoxic edema associated with TBI is now understood to result from loss of molecular homeostasis in the aforementioned post-injury milieu of metabolic, inflammatory and microvascular derangements. Specifically, ionic pump failure and aberrant activation of ion channels lead to a vicious cycle of cellular swelling, intracranial hypertension, and vascular compromise [5].

## Conclusions

To our knowledge, traumatic ischemic injury in a top of the basilar distribution has not previously been described. This intriguing finding highlights the critical need to elucidate the topography of TBI-related ischemic injury, attendant clinical outcomes, and special considerations in the context of pediatric and young adult patients.

## Data Availability

All data collected, generated and analyzed in this study are included in the published article.

## Abbreviations

CBF: Cerebral blood flow
CT: Computed tomography
ICP: Intracranial pressure
MRI: Magnetic resonance imaging
PCA: Posterior cerebral artery
TBI: Traumatic brain injury
